# Minimally Invasive Bone Expansion Using the Ridge Split Technique for Implant Site Modification: A Case Report

**DOI:** 10.7759/cureus.94913

**Published:** 2025-10-19

**Authors:** Priyanka Gupta, Mahesh Ghadage, Sumit V Bedia, Mridula Joshi, Aarti S Bedia

**Affiliations:** 1 Prosthodontics, Bharati Vidyapeeth (Deemed to Be University) Dental College and Hospital, Navi Mumbai, IND; 2 Oral Medicine and Radiology, Bharati Vidyapeeth (Deemed to Be University) Dental College and Hospital, Navi Mumbai, IND

**Keywords:** horizontal ridge augmentation, implant site modification, narrow mandibular ridge, ridge expansion, ridge split technique

## Abstract

Adequate bone volume is essential for successful dental implant placement, providing primary stability and ensuring long-term functional and aesthetic outcomes. However, horizontal alveolar ridge deficiency is a common challenge in implant dentistry, making ideal implant placement difficult and compromising the final prosthetic result. To overcome this, several bone augmentation techniques have been developed, including lateral onlay bone grafting and guided bone regeneration (GBR). While effective, these methods often require a second surgical site for graft harvesting, involve longer healing times, and carry risks such as graft resorption, membrane exposure, and infection. The ridge split technique (RST) offers a minimally invasive alternative for horizontal ridge augmentation, allowing controlled splitting and lateral expansion of the alveolar ridge. This technique often permits simultaneous implant placement, reducing treatment time, eliminating the need for additional donor sites, and enhancing patient comfort. This case report describes the use of the RST for implant placement in a narrow posterior mandibular ridge. Despite the ridge being narrow, vertical bone height was adequate. A horizontal osteotomy was performed using chisels and a microsaw, followed by gradual expansion of the buccal cortical plate while preserving the periosteum to maintain blood supply. Dental implants were placed in the same surgical procedure, achieving primary stability by engaging the apical bone. Ridge splitting in the mandible requires careful case selection and surgical precision due to the dense cortical bone, which increases the risk of fracture. Nevertheless, with a controlled, stepwise approach, predictable and stable outcomes can be achieved. This case highlights the effectiveness of the RST as a safe, reliable, and minimally invasive option for managing horizontal ridge deficiencies in implant dentistry.

## Introduction

The field of dental implantology has witnessed significant advancement over the years, but for successful implant treatment, an optimal amount of bone should be available in terms of both ridge width and height. Several studies have shown that at least 1 mm of bone width on the buccal and lingual sides of the implant is required to ensure lasting bone stability and successful implant outcomes [1].

Tooth loss triggers ongoing, unpredictable ridge resorption affecting ridge width and height due to bone remodeling. The most significant resorption typically occurs horizontally, influenced by factors such as extraction trauma, periodontal disease, or disuse. Schropp et al. (2003) observed that two-thirds of the bone width reduction occurred in the first three months [2]. Araujo and Lindhe et al., in 2005, in an animal study, observed alterations in bone width at different levels and noted that the buccal bone wall was thinner than the palatal or lingual bone wall, and the width of bone decreased toward the base [3]. These studies demonstrate that the most significant reduction in alveolar bone width occurs within the first three months following tooth extraction, which can complicate implant placement in those areas. A major challenge during implant placement is inadequate alveolar bone width [4]. Several techniques address the challenge of a narrow alveolar ridge, including onlay block bone grafting using intraoral sources, guided bone regeneration (GBR), ridge splitting or expansion, and distraction osteogenesis.

Ridge split and expansion are effective surgical interventions used to treat horizontal loss of alveolar bone [5]. This technique was introduced by Dr. Hilt Tatum in 1986. Over time, other concepts, such as the split-crest technique proposed by Summer, gained traction as professionals sought less invasive means of preparing implant sites [6]. The ridge split technique (RST) is primarily indicated in patients with a narrow alveolar ridge of at least 3 mm in width when conventional drilling may compromise the thin buccal plate. It is most effective when sufficient vertical bone height is present to expand the width while ensuring the implant's primary stability during simultaneous implant placement, which reduces overall treatment time and facilitates better outcomes for the patient [6,7]. The RST is a reliable solution for managing inadequate alveolar width in dental implantology [7]. This case report highlights the effective use of the RST to achieve adequate bone expansion for implant placement in the mandibular left first and second molar region.

## Case presentation

**Figure 1:**
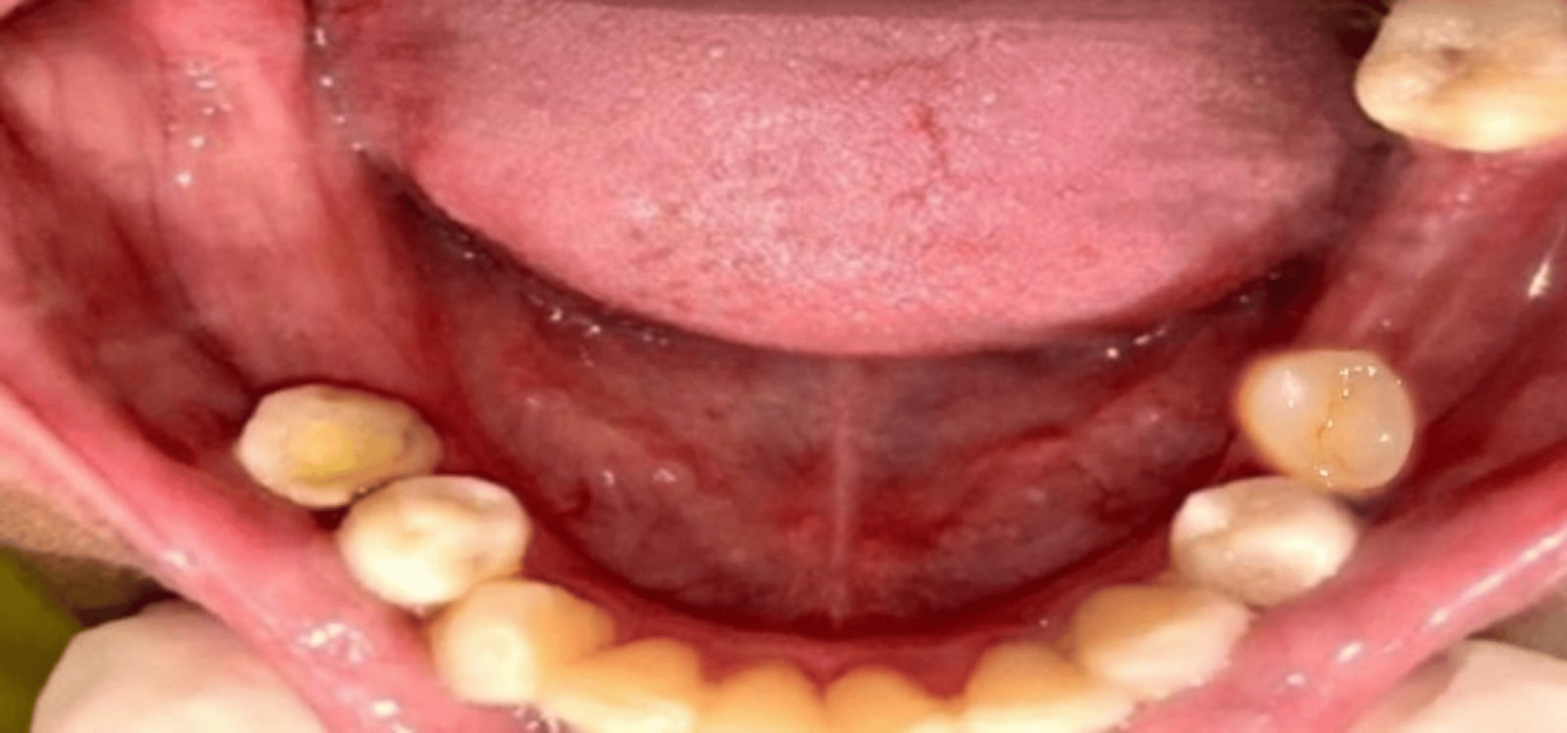
Pre-operative image

A 30-year-old female patient presented to the Department of Prosthodontics with the chief complaint of difficulty chewing. Clinical examination revealed missing teeth in her right and left lower posterior regions. The patient underwent extraction of the mandibular left molars one year ago, which resulted in decreased buccolingual width of the residual ridge (Figure 1).

**Figure 2:**
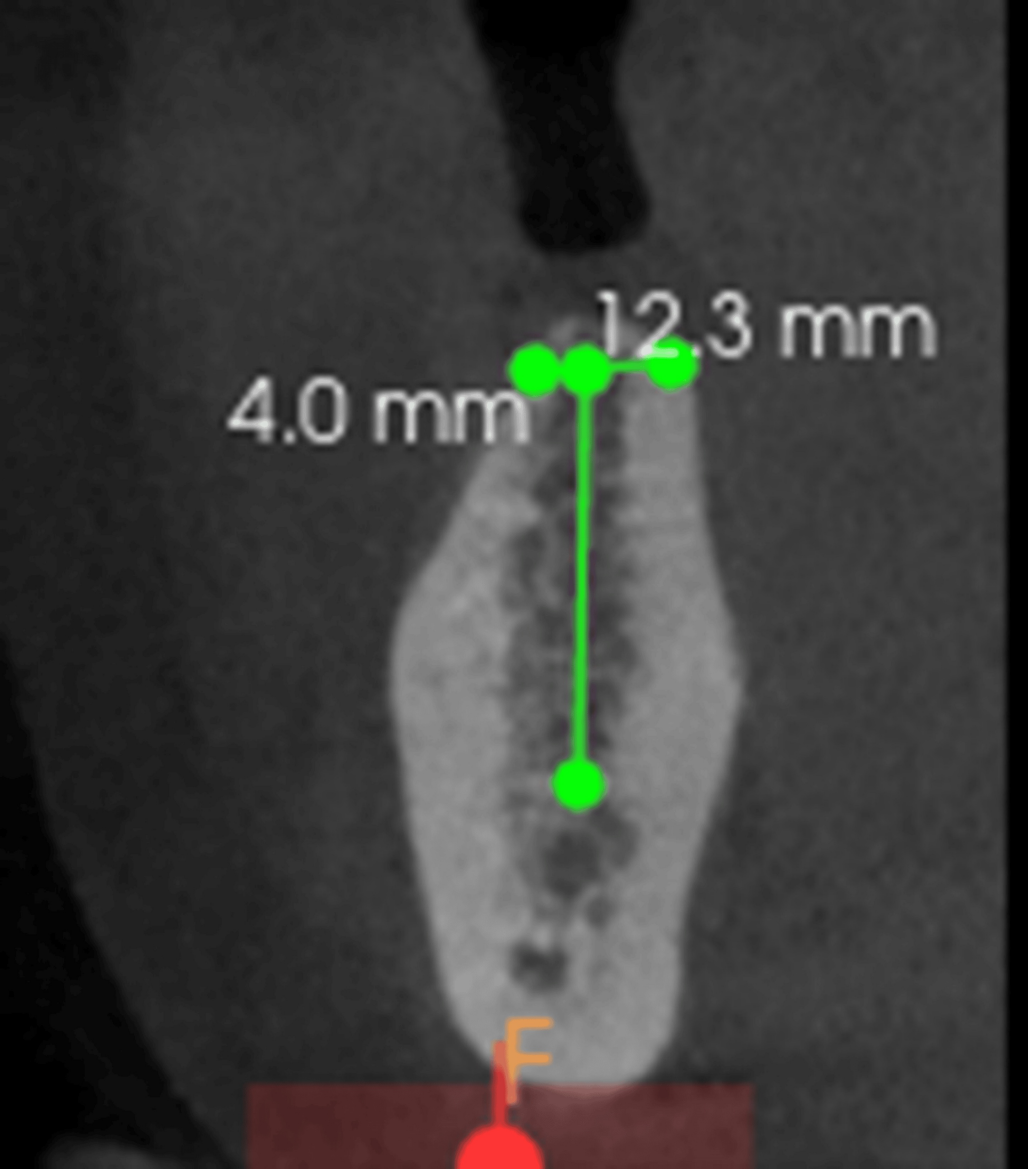
CBCT showing the width and height of the available bone CBCT, cone beam computed tomography

The patient reported no relevant systemic history. All treatment options were discussed, and the patient opted for an implant-supported prosthesis for teeth 36, 37, 46, and 47. Cone beam computed tomography (CBCT) revealed an alveolar ridge width of 4 mm, which was insufficient for implant placement. The alveolar ridge height was adequate at 12.3 mm (Figure 2).

**Figure 3:**
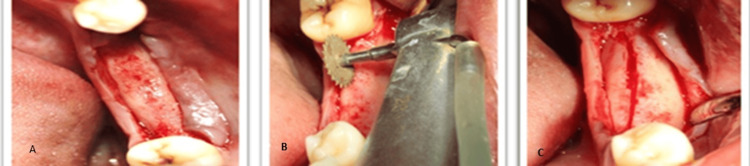
Sequence of horizontal corticotomy (A) Full-thickness mucoperiosteal flap elevation for bone exposure. (B) Horizontal corticotomy performed using a microsaw. (C) Completion of horizontal corticotomy.

Routine blood investigations revealed values within the normal reference range. Based on the preoperative assessment, a treatment plan was formulated involving the ridge split procedure with simultaneous implant placement with respect to 36 and 37, and implant placement was planned with respect to 46 and 47 with delayed loading according to the two-stage protocol. The procedure was performed under all aseptic precautions. The patient was informed about the procedure, and written consent was obtained. Following local anesthesia, a mid-crestal incision was made in the 36 and 37 regions, allowing for adequate reflection of the mucoperiosteal flap on both the buccal and palatal surfaces (Figure 3).

**Figure 4:**
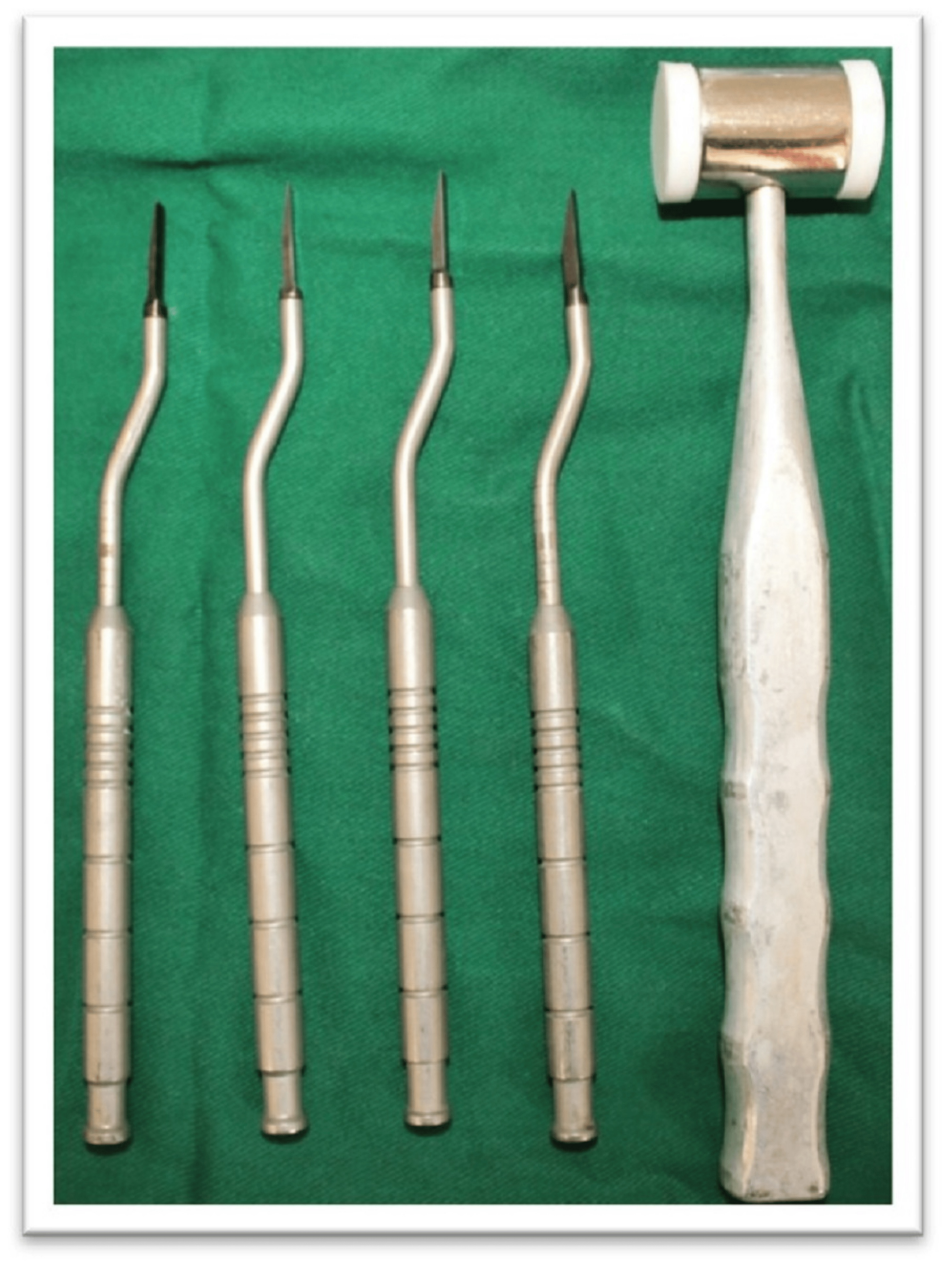
Chisel and mallet used for ridge splitting

**Figure 5:**
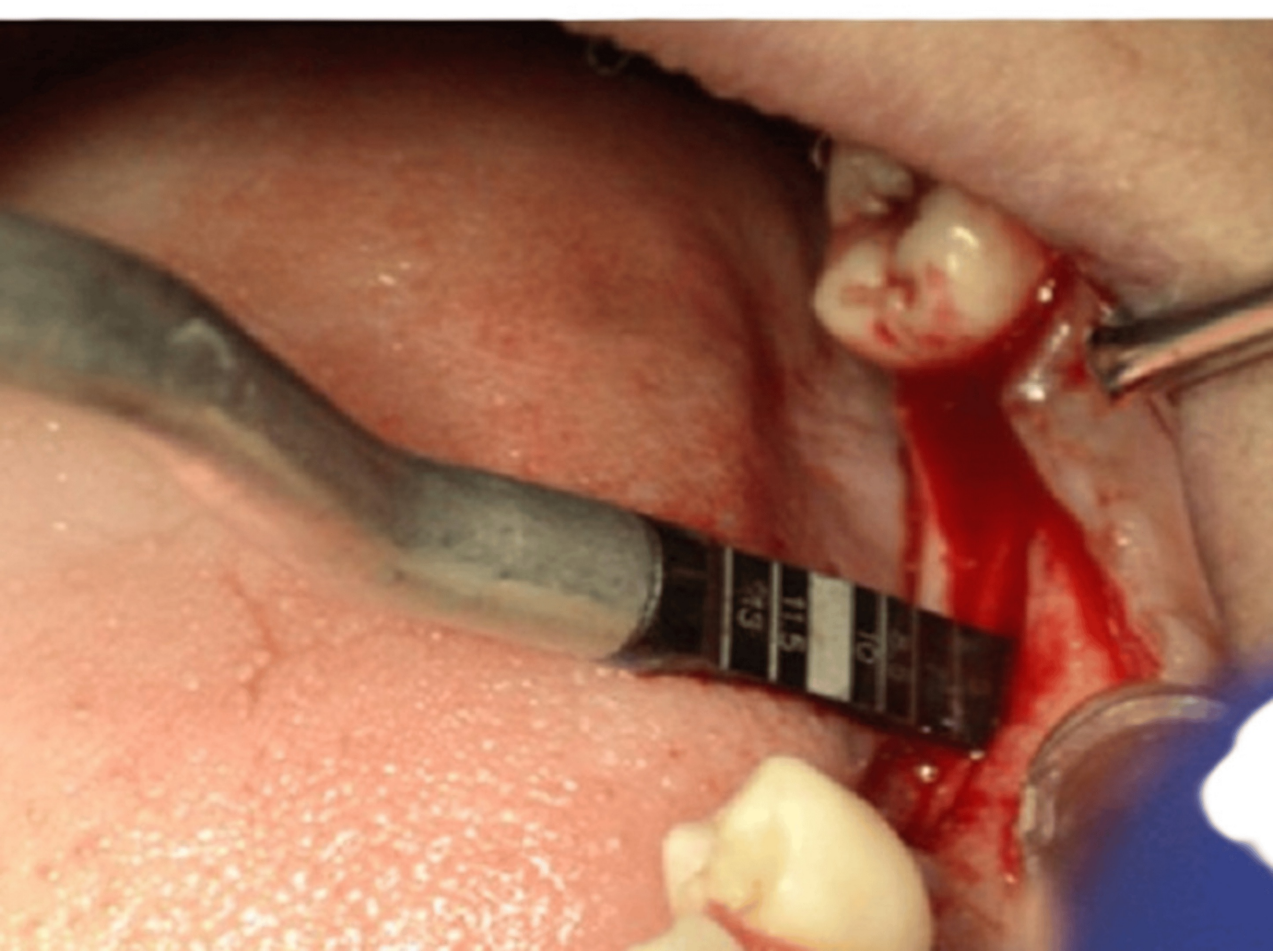
Ridge splitting being performed using a chisel

The cortical bone was curetted with a back-action chisel to remove all residual connective tissue and periosteum. Using a microsaw, a horizontal corticotomy was performed, positioned slightly lingual to the center of the edentulous ridge and spanning the entire area designated for expansion. The cut was carefully made parallel to the outer contour of the buccal plate to maintain a consistent thickness. Chisels of progressively larger sizes up to 2.5 mm in width (Figure 4) were used to gradually shift the buccal plate, resulting in a controlled greenstick fracture. A chisel was gently tapped with a hammer to initiate the cut, and the same instrument was then used as a lever to carefully separate the cortical plates. Initially, the fracture was extended to a depth of approximately 5 mm for gradual expansion, and later increased to match the implant length (Figure 5).

**Figure 6:**
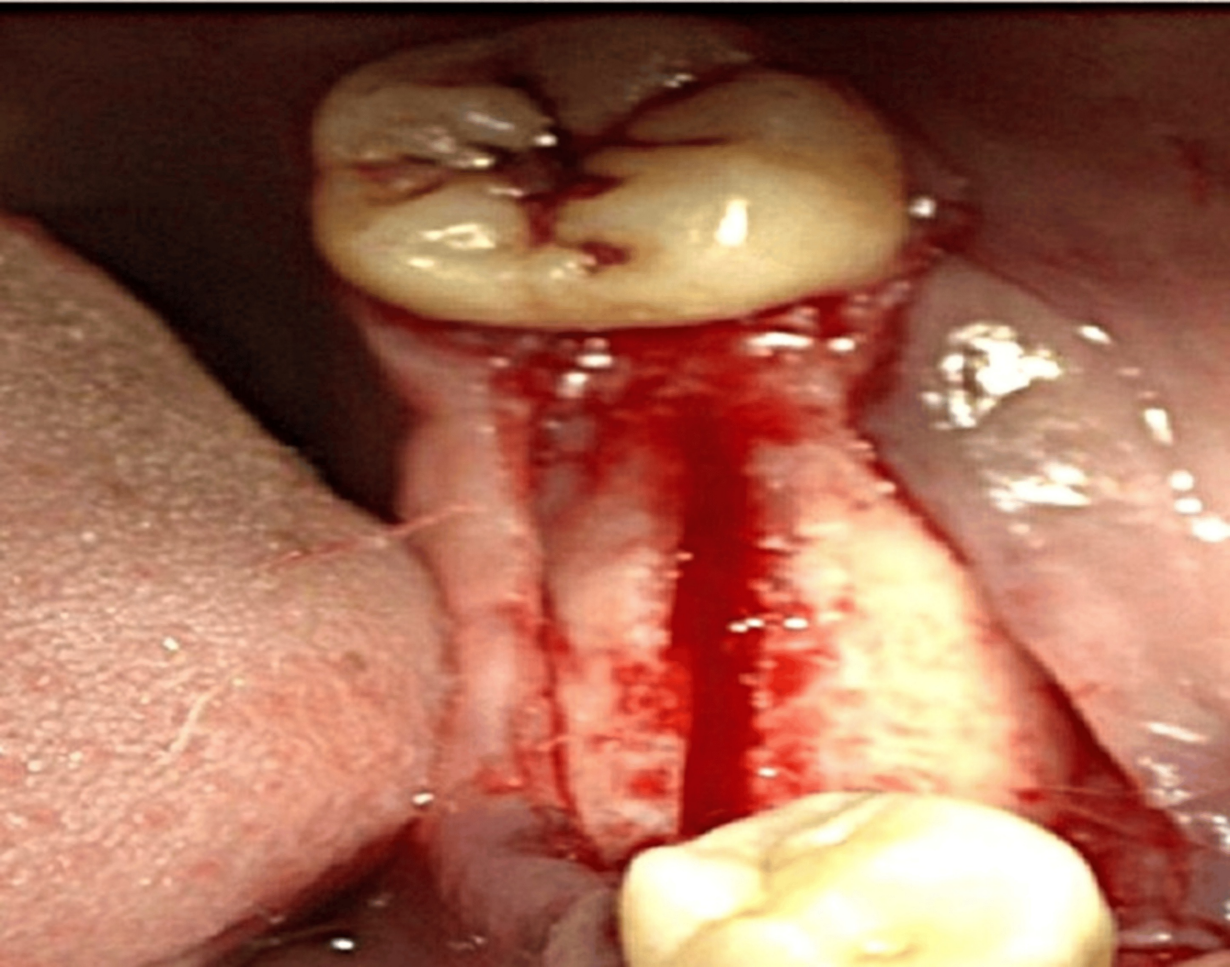
Initial ridge expansion of 5 mm achieved

**Figure 7:**
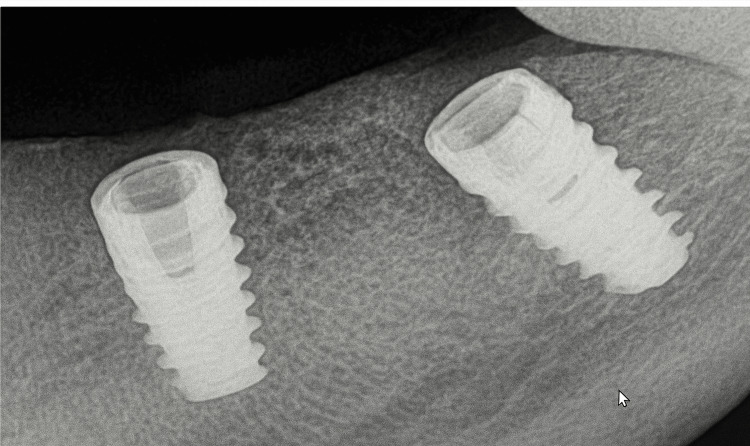
Intraoral periapical radiograph showing implants placed after ridge split procedure

**Figure 8:**
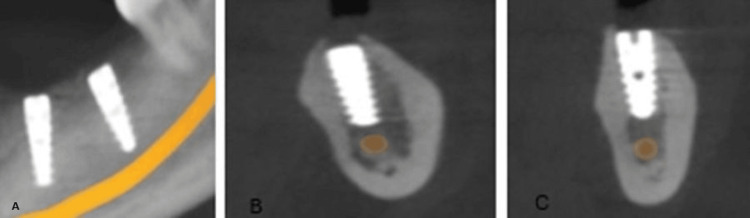
(A) Radiographic evaluation at six months. (B and C) CBCT images of the implants placed in the 36 and 37 regions, respectively CBCT, cone beam computed tomography

**Figure 9:**
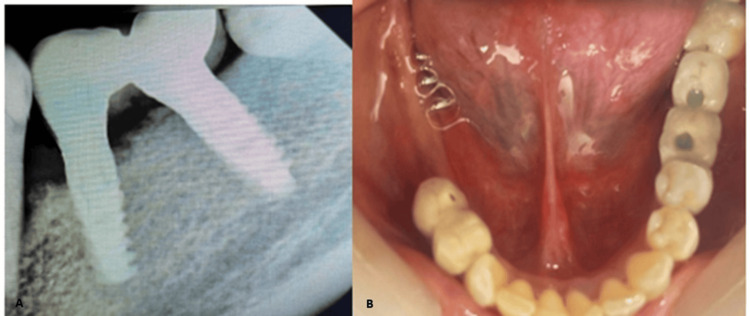
(A) Intraoral periapical radiograph showing the PFM screw- and cement-retained implant prosthesis cemented in relation to 36 and 37. (B) Intraoral occlusal view after final cementation of the prosthesis PFM, porcelain fused to metal

To ensure primary stability of the implants, a section of intact bone was preserved in the apical region. After achieving the desired ridge width of approximately 5 mm (Figure 6), two implants (ADIN 3.75×10 mm) (Figure 7) were placed in the mandibular premolar and molar region using a standard implant protocol. Grafting materials were not needed, as the remaining bone showed adequate properties for primary stability [4]. The flap was repositioned and secured with sutures, ensuring the soft tissue remained intact over the expanded ridge. Postoperative guidelines for pain management and dietary restrictions were communicated to the patient. The patient was recalled after one week for suture removal. After a healing period of six months, follow-up assessments using radiographs (Figure 8) and clinical examinations demonstrated successful osseointegration of the implants, with no signs of complications such as infection or implant failure. Implant stability was confirmed clinically using a percussion test and radiographic evaluation, both indicating successful osseointegration. Following confirmation of osseointegration, the routine prosthetic phase was carried out (Figure 9).

## Discussion

Successful dental implant placement requires adequate bone volume for stability and long-term function. However, in cases where the alveolar ridge is narrow, implant placement becomes challenging, and the final prosthetic outcome may be compromised [8]. Various bone augmentation techniques have been developed to address this issue.

Lateral onlay bone grafting and GBR are commonly used to widen the ridge. Although effective, these methods often require a second surgical site to harvest graft material, involve longer healing times, and are associated with risks such as graft resorption, membrane exposure, and infection. Studies report that bone graft resorption in lateral augmentation can range from 20% to 50%, with higher rates in the mandible than in the maxilla [6-8].

Ridge expansion can be performed in several ways. Traditional methods rely on hand tools such as chisels with mallets, osteotomes, or small surgical saws and burs. Modern techniques use powered instruments, including motor-driven expanders, threaded expanders, piezoelectric devices, lasers, or osseodensification drills. Supportive approaches include hinge-assisted ridge splits, micro-perforations to increase bone flexibility, expansion or distraction devices for gradual widening, and bone grafting or GBR with tenting. In complex cases, corticotomy-assisted procedures or segmental distraction osteogenesis may be used to manage large horizontal deficiencies [9].

The RST offers a simpler and less invasive alternative for horizontal ridge augmentation. This technique allows controlled splitting and lateral expansion of the ridge, creating sufficient space for implant placement, often in the same surgical procedure [10]. Its greatest advantages include eliminating the need for a second surgical site, reducing treatment time, lowering overall cost, and minimizing patient discomfort.

In the present case, the posterior mandibular ridge was narrow, but the ridge height was adequate. A horizontal osteotomy was carefully performed using hand instruments such as chisels and a microsaw. The buccal cortical plate was gradually expanded while preserving surrounding soft tissues and the periosteum to maintain proper blood supply, which is crucial for healing. Implants were placed simultaneously, achieving primary stability mainly by engaging the apical bone, which is critical for long-term implant success. Wound healing follows a process similar to bone fracture repair, with the gap initially filling with a blood clot that organizes and is replaced by woven bone. This immature osseous tissue subsequently matures into load-bearing lamellar bone at the implant interface. Additionally, implant surface topography may influence bone formation around implants placed during ridge splitting [11]. Ridge splitting in the mandible is more technique-sensitive due to dense cortical bone, which increases fracture risk if not performed carefully. With proper case selection and a controlled, stepwise approach, predictable outcomes can be achieved [12,13].

RST is primarily indicated for horizontal bone deficiencies. It does not provide vertical bone gain and may be more challenging in cases with single-tooth gaps or very dense bone (D1 type). Nevertheless, studies have shown good success rates, with horizontal bone gains of approximately 4 mm and implant survival rates as high as 96% after several years [6-14]. A retrospective study by Tang et al. reported high survival and success rates with minimal marginal bone loss after six months to eight years of follow-up [15].

Fracture of the buccal cortical plate during a single-stage ridge split procedure in the mandible, particularly when a full-thickness flap is reflected and the buccal bone is devascularized, can result in the segment becoming a free bone graft. In such cases, stabilization with mini screws is required, and the ridge split procedure must be delayed. Although rare, complications such as infection or excessive bleeding may occur; these are generally manageable with antibiotics, debridement, or local hemostatic agents.

To avoid neurosensory disturbances during mandibular ridge splitting, it is essential to identify the position of the mental foramen preoperatively and monitor it throughout the procedure. Other complications are uncommon [16]. The RST, when planned and executed with care, is a predictable, minimally invasive option for managing narrow alveolar ridges [17,18]. It reduces treatment time, avoids additional surgeries, and provides stable conditions for implant placement, making it a valuable tool in modern implant dentistry.

## Conclusions

The RST is a reliable solution for managing inadequate alveolar width in dental implantology. Its evolution and clinical success make it a valuable option for patients with narrow ridges. The present case highlights its practical application and favorable outcomes while emphasizing the importance of surgical skill. As research advances, further refinements will enhance its predictability and safety, ensuring its continued role in restoring function and esthetics in implant dentistry.
